# Intersecting HIV and mpox epidemics: more questions than answers

**DOI:** 10.1002/jia2.26043

**Published:** 2022-12-05

**Authors:** Nicolo Girometti, Dimie Ogoina, Darrell H. S. Tan, Anton Pozniak, Marina B. Klein

**Affiliations:** ^1^ Chelsea and Westminster Hospital NHS Foundation Trust London UK; ^2^ Infectious Diseases Unit, Department of Internal Medicine Niger Delta University/Niger Delta University Teaching Hospital Bayelsa Nigeria; ^3^ Division of Infectious Diseases St. Michael's Hospital Toronto Ontario Canada; ^4^ London School of Hygiene and Tropical Medicine London UK; ^5^ Division of Infectious Diseases and Chronic Viral Illness Service McGill University Health Centre Montreal Quebec Canada

1

Mpox was declared a Public Health Emergency of International Concern by the World Health Organization on 23 July 2022 [1], following a rapid increase in cases beginning in Europe in May 2022. By 2 November 2022, 77,264 cases had been confirmed in 109 countries, including 102 with no previously reported mpox infections [2].

Historically, mpox was largely a self‐limited zoonotic illness, affecting mostly children in rural rainforest areas in West and Central Africa [3]. However, risk factors, demographics and clinical features of the disease in Africa have been changing [3]. In the Democratic Republic of Congo, human‐to‐human transmission is increasingly reported among young adults [3], while the 2017–2018 outbreak in Nigeria predominantly affected young males in urban and semi‐urban settings without animal contacts [4]. The main modes of mpox transmission in Nigeria remain uncertain but household transmission, close contact in prisons and sexual contact may be playing roles [4, 5] heralding a change in viral and/or human behaviour that may facilitate wider transmission.

The current global outbreak is disproportionately affecting gay, bisexual and other men having sex with men (GBM). Approximately 40% of the recently diagnosed cases are among people living with HIV (PLWH) [6, 7, 8, 9, 10]. This apparent over‐representation of PLWH raises several questions. Does HIV infection modify the risk of acquiring mpox and/or its severity, or are PLWH simply more likely to be diagnosed due to better linkage with health services and knowledgeable providers? Alternatively, given the large number of men who have acquired mpox who are using HIV pre‐exposure prophylaxis (PrEP), HIV infection may be another marker of higher‐risk behaviours facilitating acquisition. It remains uncertain if mpox has become more sexually transmissible or if its introduction into large sexual networks has been the main driver of its current spread.

The concentration of mpox cases among GBM may also reinforce societal homophobia, as sexual minorities have been blamed and shamed by some for “introducing” the infection into new populations and are considered to have “deserved” the infection due to their sexual practices. Paradoxically, fears of contributing to discrimination in this way prompted some to deliberately avoid mentioning the epidemiologic association with queer communities and avoid describing mpox as a sexually transmitted infection (STI). But as HIV has taught us, silence can also cause harm. Acknowledging that mpox is spreading through sexual networks is essential to tailor knowledge dissemination strategies and culturally sensitive health services that can reach those in need. Researchers describing self‐reported sexual histories of people infected during a 2017–2018 Nigerian outbreak [5] concluded that heterosexual transmission (related to multiple partners, condomless sex and transactional sex) was likely for half of the cohort. However, they also acknowledged that criminalization of same‐sex activity, cultural pressures and religious factors may have inhibited people from self‐identifying as GBM. Providing a safe space for people to disclose sexual practices is essential, as it can enable access to other interventions, such as HIV testing, care and PrEP.

The clinical presentation of mpox in the current outbreak clearly suggests that the virus is behaving like an STI. In the early phase of the outbreak, sex‐on‐site venues and festivals were linked to transmission chains [7]. Primary lesions are frequently localized to the anogenital region (73–94%) [6, 7] or oropharynx, with other manifestations, such as lymphadenopathy (56–85%) and fever (53–72%) [6, 7, 8, 9, 10], often occurring after the onset of skin lesions [6, 7]. Painful proctitis has emerged as a serious complication and has led to hospitalizations [7]. Concomitant STIs are also frequent (15–29%) [6, 10]. Together with emerging data on higher viral loads identified in genital lesions [6], these observations support skin‐to‐skin/mucosal contact during sex as a common route for mpox transmission. Whether genital secretions (e.g. semen) can lead to the sexual transmission before lesions appear or after healing, and the duration of cutaneous/mucosal shedding of the infectious virus remain unknown—questions of utmost importance for informing recommendations for condom use.

Several studies have reported on the characteristics of people with mpox with and without HIV (Table 1). Thus far in high‐income settings, there is no clear evidence that the risk factors or clinical course differ by HIV status, except for a higher prevalence of rectal lesions at presentation among PLWH [9]. However, most cases reported have had well‐controlled HIV with high CD4 cell counts. Evidence from low‐ and middle‐income countries (LMICs) is less clear. In recent studies from Nigeria, living with HIV has been associated with a higher risk of contracting and dying from mpox (although data on HIV viral load or CD4 cell counts are lacking) [11] and with more prolonged illness, larger lesions and higher rates of both secondary bacterial skin infections and genital ulcers among hospitalized patients [12]. In Brazil, mpox patients who were PLWH were older, and more likely to have HCV coinfection, anal lesions and clinical features of proctitis [13]. Among 57 patients hospitalized with severe mpox in the United States, the majority were Black men with AIDS and untreated HIV infection (CD4<50 cells/ml) [14]. Well‐controlled studies accounting for immune status, coinfections and comorbidities are essential to understand if PLWH are at increased risk for poor outcomes, especially in LMICs where access to antiretrovirals may be limited, in order to tailor prevention and treatment interventions.

**Table 1:** Comparison in clinical differences in individuals with mpox, according to HIV status

Source	Number of PLWH *n*/total (%)	Median CD4 cell count (IQR), /μl	PLWH with viral load > 200 copies/ml *n*/total (%)	Differences between PLWH and HIV‐negative individuals with mpox
Tarín‐Vicente et al. [6]	72/181 (40)	*na* (8/72 had a CD4 cell count <500)	*na*	No differences in clinical severity or disease progression
Thornhill et al. [7]	218/528 (41)	680 (513–861)	5/190 (3)	Similar clinical presentations No differences in the frequency of hospitalization
Hoffmann et al. [8]	256/546 (47)	691 (185–1603)^a^ (7/244 had a CD4 cell count <350)	4/236 (2)	No differences in clinical presentation or hospitalization rates
Angelo et al. [9]	92/226 (41)	713 (500–885) (1/92 had a CD4 cell count <200)	7/83 (8)	PLWH more likely to present with diarrhoea and perianal rash with a higher lesion burden
Curran et al. [10]	755/1969 (38)	639 (452–831) (25/755, 3% had a CD4 cell count <200)	137/755 (18)	PLWH were more likely to report rectal pain, tenesmus and proctitis
Ogoina et al. [12]	9/40 (22)^b^	*na* (at least 7/9 had a CD4 cell count <350)	> 5/9 (>55)	PLWH were more likely to have skin rashes ≥2 cm, genital ulcers, secondary bacterial skin infection and longer duration of illness
Silva et al. [13]	109/205 (53)	527 (379–827)	8/87 (9)	PLWH were older, had higher prevalence of HCV coinfection, anal lesions and clinical features of proctitis
Miller et al. [14]	47/57 (82)^b^	*na* (40/43 had a CD4 cell count <200)	> 43/47 (>92)	Hospitalized PLWH with severe mpox were mostly not on cART and with low CD4 count. In 5/12 deaths reported, mpox was the cause of death or a contributing factor

Abbreviations: IQR, interquartile range; PLWH, people living with HIV.

^a^ Range.

^b^ All patients were hospitalized.

There are some encouraging signs that the current mpox outbreak may be declining, especially in Europe and North America where it first began. A combination of vaccination, immunity from a recent infection, increased awareness and behavioural change is likely responsible. To date, there are few cases in transgender female or heterosexual populations outside of Africa, perhaps because of specific sexual networks having a small amount of overlap. However, cases continue to rise in Africa and Brazil [15], and given the widespread nature of the epidemic and the potential to establish animal reservoirs, mpox may not be eliminated in many settings and will become another endemic STI. Several research questions thus need to be prioritized.

1. What is the level of unrecognized and ongoing sexual transmission of mpox in Africa, especially in key populations?
2. How effective and durable is vaccination at preventing infection or modifying disease course, especially after a single or reduced dose schedule?
3. Does HIV infection modify protection induced through vaccination?
4. Is treatment with tecovirimat effective at reducing symptoms, particularly those most troublesome such as proctitis, and will treatment speed time to recovery and reduce onward transmission?
5. Can treatment resistance develop, and will HIV infection modify resistance risk?

Randomized controlled trials are needed to answer these questions, and some are underway (NCT05534984, NCT05559099, NCT05534165 and Platinum). Such trials should be conducted in both low‐ and high‐income settings and should plan for and/or combine data for subgroup analyses on PLWH. Successful interventions emerging from them should benefit all settings, including LMICs, where mpox remains endemic.

Combating stigma, discrimination and providing person‐centred care have been core to the HIV response and are essential for appropriate clinical and public health services for all evolving pandemics, including mpox. Established principles of “greater involvement/ meaningful engagement of people living with HIV/AIDS (GIPA/MEPA)” highlight the public health imperative of privileging the voices of those most affected and allowing all affected communities to co‐lead in all aspects of the epidemic response and to guide future research.

## COMPETING INTERESTS

DHST's institution has received support from Abbvie and Gilead for investigator‐initiated research, and support from Glaxo Smith Kline for industry‐sponsored clinical trials. AP served on advisory boards and received educational grants from ViiV, Gilead, MSD and Janssen. MBK reports grants for investigator‐initiated studies from ViiV Healthcare, AbbVie, Merck, and Gilead; and consulting fees from ViiV Healthcare, Merck, AbbVie, and Gilead. NG and DO have no competing interests to disclose.

## AUTHORS’ CONTRIBUTIONS

All authors contributed to the conceptualization of the manuscript, were involved in drafting the manuscript and critically revised the manuscript. All authors reviewed and approved the final version of the manuscript.
